# Brain–Computer Interface-Robot Training Enhances Upper Extremity Performance and Changes the Cortical Activation in Stroke Patients: A Functional Near-Infrared Spectroscopy Study

**DOI:** 10.3389/fnins.2022.809657

**Published:** 2022-04-08

**Authors:** Lingyu Liu, Minxia Jin, Linguo Zhang, Qiuzhen Zhang, Dunrong Hu, Lingjing Jin, Zhiyu Nie

**Affiliations:** ^1^Department of Neurorehabilitation, Shanghai Yangzhi Rehabilitation Hospital, Shanghai Sunshine Rehabilitation Center, School of Medicine, Tongji University, Shanghai, China; ^2^Department of Neurology, Tongji Hospital, School of Medicine, Tongji University, Shanghai, China

**Keywords:** stroke rehabilitation, brain–computer interface, upper extremity, functional connectivity, functional near-infrared spectroscopy

## Abstract

**Introduction:**

We evaluated the efficacy of brain–computer interface (BCI) training to explore the hypothesized beneficial effects of physiotherapy alone in chronic stroke patients with moderate or severe paresis. We also focused on the neuroplastic changes in the primary motor cortex (M_1_) after BCI training.

**Methods:**

In this study, 18 hospitalized chronic stroke patients with moderate or severe motor deficits participated. Patients were operated on for 20 sessions and followed up after 1 month. Functional assessments were performed at five points, namely, pre1-, pre2-, mid-, post-training, and 1-month follow-up. Wolf Motor Function Test (WMFT) was used as the primary outcome measure, while Fugl-Meyer Assessment (FMA), its wrist and hand (FMA-WH) sub-score and its shoulder and elbow (FMA-SE) sub-score served as secondary outcome measures. Neuroplastic changes were measured by functional near-infrared spectroscopy (fNIRS) at baseline and after 20 sessions of BCI training. Pearson correlation analysis was used to evaluate functional connectivity (FC) across time points.

**Results:**

Compared to the baseline, better functional outcome was observed after BCI training and 1-month follow-up, including a significantly higher probability of achieving a clinically relevant increase in the WMFT full score (ΔWMFT score = 12.39 points, *F* = 30.28, and *P* < 0.001), WMFT completion time (ΔWMFT time = 248.39 s, *F* = 16.83, and *P* < 0.001), and FMA full score (ΔFMA-UE = 12.72 points, *F* = 106.07, and *P* < 0.001), FMA-WH sub-score (ΔFMA-WH = 5.6 points, *F* = 35.53, and *P* < 0.001), and FMA-SE sub-score (ΔFMA-SE = 8.06 points, *F* = 22.38, and *P* < 0.001). Compared to the baseline, after BCI training the FC between the ipsilateral M_1_ and the contralateral M_1_ was increased (*P* < 0.05), which was the same as the FC between the ipsilateral M_1_ and the ipsilateral frontal lobe, and the FC between the contralateral M_1_ and the contralateral frontal lobe was also increased (*P* < 0.05).

**Conclusion:**

The findings demonstrate that BCI-based rehabilitation could be an effective intervention for the motor performance of patients after stroke with moderate or severe upper limb paresis and represents a potential strategy in stroke neurorehabilitation. Our results suggest that FC between ipsilesional M_1_ and frontal cortex might be enhanced after BCI training.

**Clinical Trial Registration:**

www.chictr.org.cn, identifier: ChiCTR2100046301.

## Introduction

Stroke is one of the most prevalent pathologies which causes devastating consequences in most of the survivors worldwide (Benjamin et al., 2017). Rehabilitation is often operated early to minimize disability and to improve the quality of patients' daily life. Most patients reach a functional plateau, especially 6 months after the onset. Previous research findings have suggested that some neural plasticity, which may hamper functional recovery, might occur, and gradually persist during this plateau, has been approaching (Miller et al., 2013). This promotes many uses of alternative, non-conventional treatments such as constraint-induced movement therapy (Varkuti et al., 2013), robot-assisted movement therapy (Pinter et al., 2013), and brain–computer interface (BCI) (Tariq et al., 2018). Recently, with the advancements in neurotechnology, BCI has become the emergence in stroke rehabilitation. It is an innovative intervention that records and decodes neural signals by real-time electroencephalogram (EEG) and transfers them into digital signals. Then assistive devices, such as prostheses or robots, can be triggered by specific signals. The EEG-based BCI has emerged as a potentially effective therapeutic scheme in motor recovery in the chronic stroke stage. Neural signals are detected and inputted to provide real-time feedback, which effectively enables patients to modulate their brain activity (Cervera et al., 2018). Several research studies have recently provided evidence that BCI promotes functional recovery in upper limb or hand function (Ang et al., 2015a; Kasashima-Shindo et al., 2015), although others have found no changes when compared to robots in the chronic stage (Ang et al., 2014).

In order to increase the BCI performance accuracy, stroke survivors carry out motor imagery (MI) exercises or motor watching during EEG recording (Cervera et al., 2018). MI induced the decoded brain oscillations, which is used to trigger a robotic device to reproduce the real movement with the paretic limb (Irimia et al., 2016). These types of multimodal feedback, including visual, haptic, and kinesthetic feedback, provide a closed-loop feedback system for patients. Numerous neurophysiological studies have demonstrated the effect of MI on motor function and neuroplasticity. In these findings, it has been shown that the MI-based BCI rehabilitation system could activate the primary motor cortex (M_1_) and other brain structures involved in motor planning and control of voluntary movements (Ramos-Murguialday et al., 2013; Ono et al., 2014).

Previous studies have demonstrated that motor recovery has been related to modulating changes in neuroplasticity in the adult human brain (Wander et al., 2013). A growing number of studies have shown that specific regional activation in the cortex and motor recovery after stroke are closely related processes (Grefkes et al., 2008). The changes beyond the motor network after BCI is unclear, especially the functional connectivity (FC) or neural reorganization of cortical regions between the motor network and the sensor network. FC and basic reorganization changes of the motor recovery can be investigated using many neuroimaging techniques, including functional magnetic resonance imaging (fMRI), electroencephalography (EEG), and functional near-infrared spectroscopy (fNIRS) (Sun et al., 2017; Yang et al., 2019).

Functional near-infrared spectroscopy is an emerging technology that measures the concentration changes in oxy-Hb and deoxy-Hb caused by brain activity. It has been known that the requirement for oxygen during brain activation generates the dilatation of arterioles and capillaries, which is called neurovascular coupling (NVC) during the process of the local neural activity and metabolism between neurons and other tissues. Based on the NVC mechanism, cerebral oxygenation fluctuation signals can be recorded by fNIRS, and the coherence and phase-locking value (PLV) be calculated, by which the FC of the brain networks could be assessed (Briels et al., 2020). By performing the PLV, we could identify the consistent phase differences that indicate high phase synchronization. The better the consistency of the hemodynamic changes in a specific frequency domain in different cortical regions, the stronger the connectivity of the neural activity between these regions will be. Previous studies have provided strong evidence that stroke plays a causal role in impairing cerebral blood flow (CBF) regulation during brain activation, which depends on NVC. Real-time adjustment of CBF to neuronal activity *via* NVC has an essential role in the maintenance of normal brain function (Tarantini et al., 2017). Simultaneously, this response reduces the resistance of the vascular bed to guarantee adequate cerebral blood perfusion to activate neurons. The development of fNIRS has greatly advanced the understanding of the underlying behavior of neural mechanisms and remodeling after brain lesions in humans. It provides real-time sensitivity of the brain's oxygenation state. Additionally, compared with fMRI, fNIRS measurement can be taken in an upright position with a higher temporal resolution (~10 Hz) and in a task state without physical restraint. Several studies using fNIRS have successfully observed FC during both the rest and the task state in healthy volunteers (Lu et al., 2010). Recently, this method has gradually become a well-established neuroimaging tool in scientific studies.

In this study, we explored the efficacy of rehabilitation in upper extremity motor recovery after BCI training and the neuroplastic changes in cortical organization. In this single pre-post intervention group study, we designed a task-related and clinical setting by using fNIRS to evaluate whether the brain activation for M_1_ can be assessed routinely after BCI training in chronic stroke. We hypothesized an improvement in motor performance and a strengthening of the FC between ipsilateral M_1_ and the frontal cortex after BCI training.

## Materials and Methods

### Participants and Study Design

The study was conducted and approved by the Human Ethics Committee of Shanghai Yangzhi Rehabilitation Hospital (#SBKT-2021-044), at which all participants had completed inpatient rehabilitation and had received standard medical care and traditional rehabilitation for 4 weeks, which consisted of routine physiotherapy and occupational therapy focused on rehabilitation of functional transfer. Any activities involving arm and hand movements were avoided. All participants provided written informed consent before the beginning of the study. The inclusion criteria for these patients were as follows: (1) First-time onset stroke patients diagnosed by computed tomography or brain MRI (poststroke time ≥6 months); (2) stable neurological status with unilateral residual hemiplegia; (3) functional restriction in the upper extremities and Brunnstrom stage ≥II; (4) right-handed individuals, who were confirmed by the Edinburgh Handedness Inventory; (5) with the ability to understand the therapists' direction and MMSE score ≥21 according to the education level. Those excluded were (1) with medical instability such as heart/respiratory failure, deep venous thrombosis, acute myocardial infarction, non-compensated diabetes, active liver disease, or/and kidney dysfunction; (2) with the severe cognitive disorder or/and MMSE score <21 that the patient cannot follow and perform tasks; (3) with severe aphasia; (4) with limitation of passive range of motion in the paretic upper limb (dorsal wrist flexion < 20°, limitation of elbow flexion > 30°, and shoulder abduction < 60°); (5) with upper limb muscular-skeletal diseases such as a fracture; (6) with a mental illness history or taking any antipsychotic drugs that are not suitable for this study; and (7) those who had received antispastic therapy (including any antispastic medicine or botulinum toxin injection) within the 6 months prior to the study.

**Figure 1:**
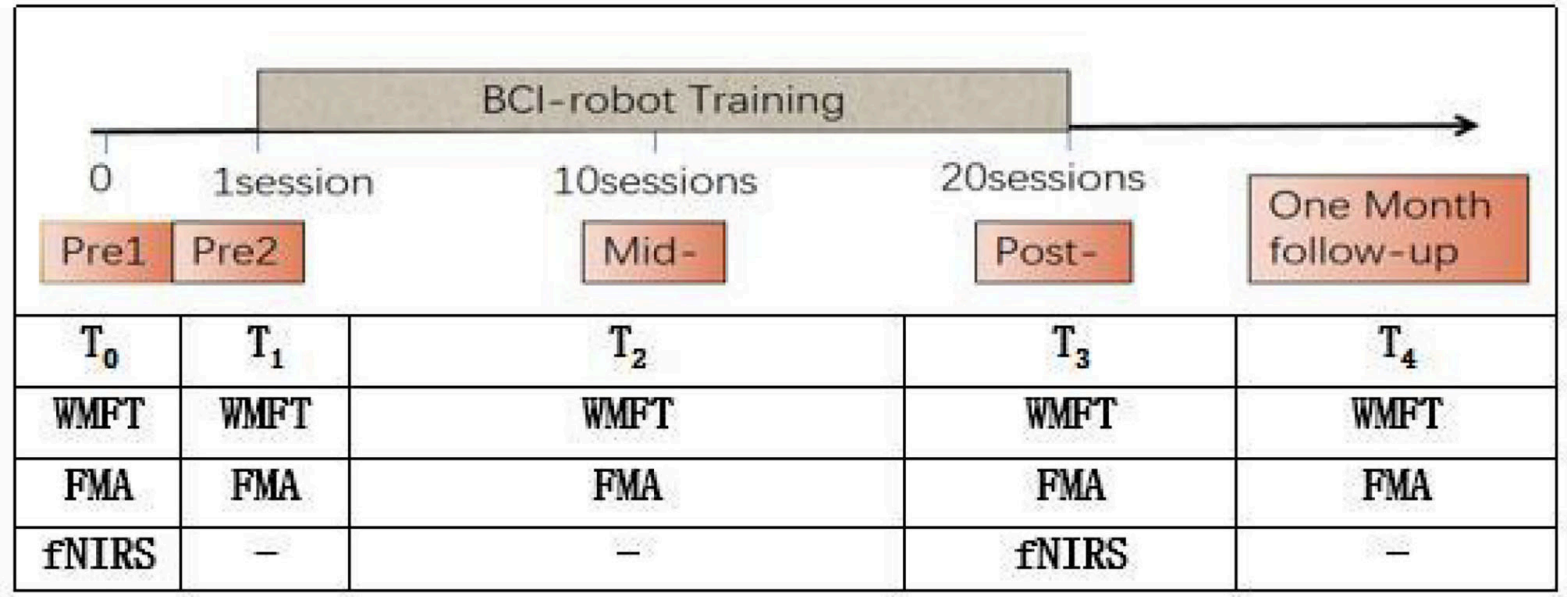
Timing of assessments.

Patients accepted the BCI-robot training system. Each patient performed 20 sessions (one session per day and 5 days per week) and followed up after 1 month. The sessions were conducted every day except weekends and holidays. Functional assessments were performed at five points, namely, pre1, pre2, mid-, post-, and 1-month follow-up (FU). fNIRS assessments were measured at two time points, namely, pre1 and post BCI training. Two qualified therapists performed all training and another researcher in the team conducted the evaluations. Pre1 and Pre2 were scheduled within 3 days before the intervention, respectively, while mid-, post-, and 1-month FU were carried out, respectively, after 10 sessions of training, 20 sessions of training, and 1 month after the intervention (see Figure 1).

### Participants' Baselines

A total of 21 hemiplegic patients were recruited and 18 completed this study. Altogether, there were 14 men and 4 women (mean age = 45.33 years, SD = 15.07). The stroke duration ranged from 6 to 20 months [median time = 6.5 months, IQR = 6–8 months]. Notably, nine patients had a stroke in the right hemisphere (50%), and 9 in the left hemisphere (50%). None of them had brain-stem involvement (Table 1).

**Table 1:** Demographic, clinical, and neuropsychological characteristics of the patients.

**Number**	**Age**	**Gender**	**Hemisphere lesion**	**Type of lesion**	**Days after stroke (m)**	**BI**	**Brunnstrom**
1	40	M	L basal ganglia	IS	8	98	II
2	53	M	L lateral ventricle	IS	6	93	IV
3	72	M	L MCAO	IS	6	85	IV
4	35	M	L internal capsule	H	6	92	III
5	43	M	L basal ganglia	H	6	92	III
6	23	M	L corona radiate	H	8	77	II
7	28	M	L basal ganglia	H	6	69	II
8	29	F	L basal ganglia	H	9	78	II
9	73	M	R lateral ventricle	IS	7	73	II
10	39	M	R basal ganglia	H	6	74	II
11	39	M	R MCAO	IS	9	67	II
12	51	F	R corona radiate	H	6	73	II
13	29	M	R hemisphere	H	20	79	II
14	39	M	L basal ganglia	H	7	59	II
15	44	M	R temporal lobe	H	6	61	II
16	67	F	R internal capsule	H	6	77	II
17	55	F	R corona radiate	H	7	98	II
18	57	M	R basal ganglia	IS	7	71	III

*M, male; F, female; R, right; L, left; MCAO, middle cerebral artery occlusion; BI, Barthel index; IS, ischemic; H, hemorrhagic*.

### Motor and Cognitive Assessments

Functional and behavioral scales were performed to measure the motor improvements. The Wolf Motor Function Test (WMFT, score from 0 to 75) was used as the primary outcome measure, while Fugl-Meyer Assessment (FMA, score from 0 to 66), its wrist and hand (FMA-WH) sub-score, and shoulder and elbow (FMA-SE) sub-score served as secondary outcome measures. To detect the baseline stability, the clinical assessments were done two times in 3 days in the beginning of the training. The different scores reflect different extents of impairment in upper limb functions. The lower scores correspond to greater impairment. The Brunnstrom stage (from stage I to stage VI) was also used to measure baseline impairment for this clinical trial. The activities of daily living recovery were evaluated using the Barthel index (BI, score from 0 to 100), which includes 10 items (a score of 100 corresponding to complete independence). In addition, we used the Mini-Mental State Examination (MMSE, score from 0 to 30) for cognitive assessment. Higher scores indicate better cognitive function, and a score below 25 points is considered to be abnormal. Those above 21 points (according to the education level) are anticipated.

### BCI-Robot System Description

**Figure 2:**
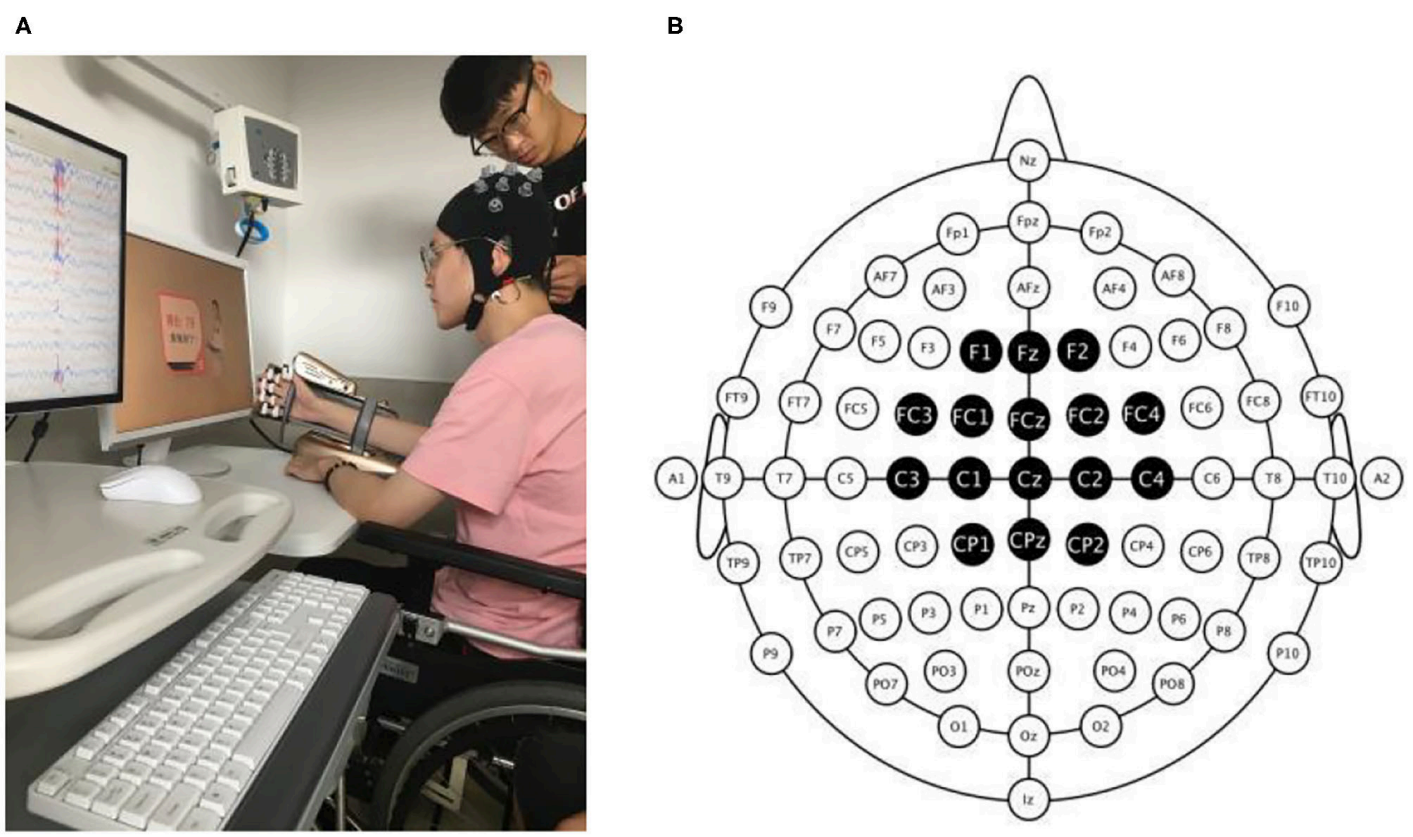
Photograph of the BCI-robot training system. **(A)** The whole system includes an EEG amplifier collecting real-time EEG, a PC processing EEG signal providing visual and auditory feedback, and a triggered-robot hand supporting sensory and movement feedback. **(B)** The montage of real-time EEG electrodes. There were 16 active electrodes placed on the frontal and parietal cortex.

MI-based BCI-robot training system (RHB-III with 16 EEG channels; Shenzhen Rehab Medical Technology Co., Ltd., China) is shown in Figure 2A. The whole system consisted of the collection system of real-time EEG signals, a central processing control algorithm, and a manus robot feedback. In all patients, EEG was recorded using 16 active electrodes within the 10–20 system of electrode locations over the frontal and parietal regions. Recording locations were channel positions F1, Fz, F3, FC3, FC1, FCz, FC2, FC4, C3, C1, Cz, C2, C4, CP1, CPz, and CP2 (Figure 2B). The real-time EEG signals were amplified and processed by the central processing control algorithm. Video clips on the computer screen were played to guide the participants to execute MI tasks. An exoskeleton robot hand was used to help the paretic hand perform the real movement in grasping/opening tasks. A mu event-related desynchronization (ERD) (score from 0 to 100) was displayed to provide real-time visual feedback. Participants could modify their MI strategy to achieve MI-triggered robotic feedback more successfully (Sun et al., 2017).

Electroencephalogram signals from the C3 and C4 electrodes were used for BCI control. A voice clue was used before each trial began to help the participant on the MI task. The system presented the instruction on the screen after 2 s. Then, a text and voice cue of “hand grasp”/“hand open” was displayed and lasted for 2 s. Meanwhile, a hand movement video clip with a duration of 6 s was then displayed. Participants can watch the video and perform the same MI processing in their brains. EEG signal measurements were collected with unipolar Ag/Ag-Cl electrode channels and digitally sampled at 256 Hz with a resolution of 22 bits for voltage ranges of ±130 mV. In terms of ensuring the transmission impedance was below 1 kΩ, all electrodes were immersed in saltwater for preparation.

In this study, the paretic hand was strapped to the manus robotic exoskeleton. Participants were instructed to watch the actions displayed in the video and guided to imagine they were performing the same movement with the paretic hand. According to the real-time EEG signals, the calculation of the mu ERD score was conducted. When the score was above 60, the robot would be triggered and assist the paretic hand to accomplish the grasp/open task for the next 3 s. But if the mu ERD score was below 60, the robot would not be triggered to move, which was considered a failed trial. The mu ERD score was then shown for 2 s. Participants were encouraged to instruct MI until successful or unsuccessful detection was indicated on the video screen. If MI was successfully detected, visual and movement feedback were provided by the robot through the real movement of the paretic hand. The BCI-robot therapy session included 4 runs of 40 trials each, for a total of 160 trials, and an interrun break of 3 min. It took about 40–50 min for each BCI training session in total.

### Functional Near-Infrared Spectroscopy

**Figure 3:**
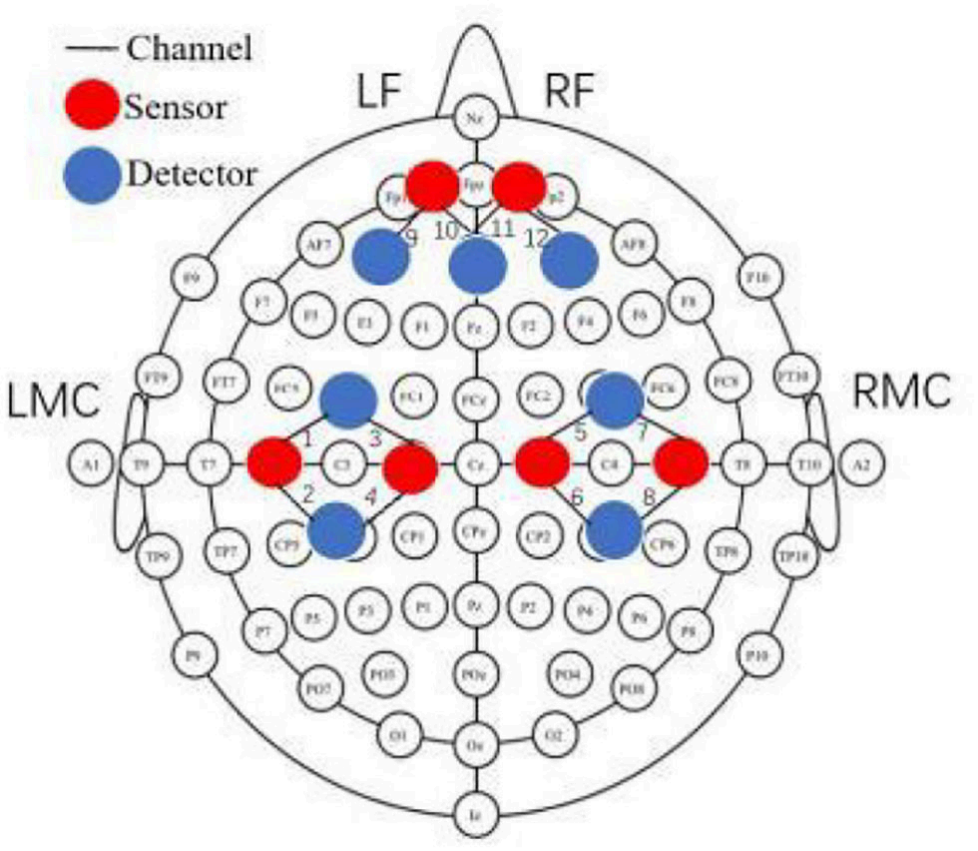
Functional near-infrared spectroscopy (fNIRS) 12-channel montage configuration. The probes were located over the frontal cortex (left frontal cortex and right frontal cortex near FP_Z_), the primary motor cortex (M_1_) (left M_1_ and right M_1_). The red circles represent the positions of sensors (S) and the blue circles represent the positions of detectors (D). The line between them is the channel. LMC, left M_1_ cortex; RMC, right M_1_ cortex; LF, left frontal cortex; RF, right frontal cortex.

In this study, we utilized the multichannel fNIRS system (NirScan, NirScan-6000, USA) to measure the changes in concentration of oxygenated hemoglobin (oxy-Hb) and deoxygenated hemoglobin (deoxy-Hb). Channels between each transmitter and receiver were placed according to the international 10–20 system, in which the distance was set at 3–5 mm. The sampling rate of the NIRS system was set to 10 Hz, and the wavelengths used were 740 and 850 nm. The probe layout consisted of 12 channels (30 mm spacing interval). The channel montage configuration of the NIRS probe set is shown in Figure 3. The optodes were positioned over the primary motor cortex area (LMC: L1–L4; RMC: R5–R8) and the frontal cortex area (LF: L9 and L10; RF: R11 and R12). Prior to each recording, a NIR gain quality check was performed to ensure data acquisition was moderate, namely, neither under-gained nor over-gained. To place the probes in a fixed position on the scalp, the participant's head was covered with a cap and fixed with a trap to adjust and fixate the transmitters and receivers. In order to attain maximum efficiency of light coupling to the tissue, hairs were carefully swept away to ensure the optodes touched the participant's skin tightly (see Figure 3).

**Figure 4:**
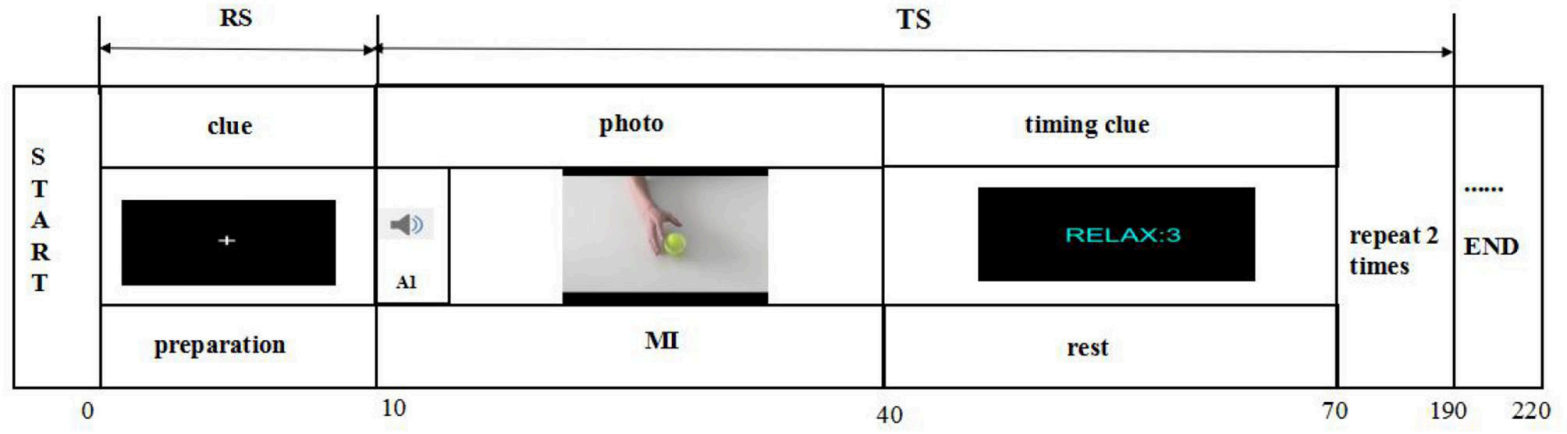
Illustration of fNIRS testing. There are two states of fNIRS testing, namely, the rest state (RS) and the task state (TS). The RS is 10 s, and the TS is a 180-s grasping task. The grasping task consists of three trials, and each trial is followed by a 30-s intertrial break.

As shown in Figure 4, the experimental design in the fNIRS testing comprised two periods, namely, the resting state (RS) and task state (TS). The RS required the subjects to sit quietly for 10 s to become ready. The participants were requested to close their eyes, relax, and avoid any movements except those needed for the motor tasks to avoid affecting the blood oxygen data. Before measurement, participants were taught to rehearse the motor task to comprehend the task instructions. For the TS, the participants were instructed to perform a repetitive three-time grip-and-rest task for a total of 180 s by using a dynamometer as accurately as possible. During this TS, the relax duration was set to 30 s for NIRS signals reaching the baselines. Then, the participants were required to sit for at least 30 s as the RS. The total trial time of one session was thus 220 s.

Functional near-infrared spectroscopy data processing was operated as follows. First, a proficient expert made a preliminary examination of the raw data, and the signals with poor quality were marked and removed. Second, signals containing motion artifacts were labeled and excluded for calculating task-related changes. Subsequently, further analysis on only the oxy-Hb and deoxy-Hb data of non-marked channels covering functionally involved areas, namely, the four regions of interest (ROIs), was performed. Third, according to the modified Beer-Lambert law, data processing was made using a MATLAB script. A high pass filter with a cutoff frequency at 0.01 Hz was used to make the baseline removal, such as the physiological signals, and the low pass filter with a cutoff frequency at 0.8 Hz was applied to the signals. Transfer function models were applied for artifact reduction of the systemic influences (Bauernfeind et al., 2014). Then the baseline correction on each trial was performed to make the task state stable and the baseline signal began at approximately zero. The average time for hemodynamic response function (HRF) in each ROI was measured for each participant over each recorded trial at task state. The averaged amplitude of each HRF was also calculated for statistical analysis. FC was calculated in terms of both time and frequency. We utilized the correlation approach to estimate the strength of the pairwise Pearson's correlation between ROIs for the time aspect (Pannunzi et al., 2017). In terms of frequency, coherence and phase-locking value (PLV) were applied to analyze the level of synchronization of the fNIRS signals, which indicated the stability of the phase difference between the two time series (Briels et al., 2020). We performed Welch's averaged, modified periodogram method to calculate the squared coherence between ROIs. All connectivity matrices were made to be Fisher's *z*-transformed for statistical analysis (Arun et al., 2020).

### Statistical Analysis

Measurement data were expressed by mean and standard deviation. All data analyses and statistics were performed in MATLAB. All data were tested for normal distribution using Shapiro–Wilk test. The mean comparison after the intervention was performed using a one-sample *t*-test or Kruskal–Wallis test. Repeated measures ANOVA with the Bonferroni *post-hoc* test was utilized to measure the differences in the clinical assessments at five different time points (two pre-assessments, a mid-assessment, a post-assessment, and a 1-month FU assessment). Repeated measures of ANOVA were also used to compare FC matrices at baseline and after BCI training. Pearson correlation analysis was utilized between the FC. The statistically significant level was 0.05 in this study.

## Results

### Functional Improvement After BCI-Robot Therapy

Totally, 21 participants were recruited and received the screen learning control. Two participants were unable to achieve control over the BCI, and one was discharged earlier than expected due to non-medical reasons. Finally, 18 participants completed 20 sessions of BCI-robot training and a 1-month FU, with all participants completing the clinical assessments and fNIRS measurements. No adverse effects were reported.

### Clinical Functional Assessments

Motor improvements measured by clinical scores, including the WMFT and FMA-UE scores, are summarized in Table 2. Significant increases were observed in the WMFT full score (*P* < 0.001, ΔWMFT score = 12.39 points, *F* = 30.28, one-way ANOVA with the Bonferroni *post-hoc* test), FMA full score (*P* < 0.001, ΔFMA-UE = 12.72 points, *F* = 106.07, one-way ANOVA with the Bonferroni *post-hoc* test), and WMFT completion time score (*P* < 0.001, ΔWMFT time = 248.39 s, *F* = 16.83, one-way ANOVA). As depicted in Table 2, a statistically significant mean FMA-UE increase of 12.72 points has been observed. Importantly, as recommended in the literature (Page et al., 2015), the minimal clinically important difference (MCID) for the FMA-UE scale is accepted to be a 5-point increase in chronic stroke survivors. Table 2 lists all the clinical scores measured in this study (i.e., means and 95% confidence intervals of each clinical assessment as well as the one-way ANOVA probabilities for evaluation with respect to the assessment sessions). As shown in Table 2, FMA-WH sub-score ΔFMA-WH = 5.6 points, *F* = 35.53, and *P* < 0.001) and FMA-SE sub-score (ΔFMA-SE = 8.06 points, *F* = 22.38, and *P* < 0.001) have also increased significantly, respectively.

**Table 2:** Means and 95% confidence intervals for each assessment at five time points, as well as the probabilities and *F*-value of the statistical analyses.

	**Pre1-**	**Pre2-**	**Mid-**	**Post-**	**1-month FU**	**One-way ANOVA**	
**Evaluation**	**Mean (95% confidence interval)**					***P*-value**	***F*-value**
**WMFT**							
Score	24.89 (16.97–32.81)	26.22 (18.22–34.23)	30.22 (21.03–39.41)	34.17 (24.63–43.70)	37.28^***^ (27.14–47.41)	<0.001	30.38
Time	1,076.44 (856.41–1,296.48)	1,040.39 (825.35–1,255.43)	986.78 (768.13–1,205.43)	899.61 (676.29–1,122.93)	828.06^***^ (619.152–1,036.96)	<0.001	16.83
**FMA-UE**							
Full score	28.06 (21.26–34.85)	29.61 (22.937–36.29)	32.89 (26.25–39.53)	36.50 (29.75–43.25)	40.78^***^ (34.00–47.56)	<0.001	106.07
Wrist/hand	9.83 (7.05–12.62)	9.55 (7.21–11.65)	11.83 (8.78–14.89)	13.78 (10.67–16.89)	15.44^***^ (12.38–18.51)	<0.001	35.53
Shoulder/elbow	17.67 (13.10–22.24)	18.24 (12.41–21.43)	21.83 (17.11–26.56)	22.50 (17.86–27.14)	25.72^***^ (21.07–30.38)	<0.001	22.38

*Significant levels are demonstrated as*

*** *for p < 0.001*.

*WMFT, Wolf Motor Function Test; FMA, Fugl-Meyer Assessment; Pre1, first pre-training assessment; Pre2, second pre-training assessment; Mid-, ten-training assessment; Post-, post-training assessment; 1-month FU, 1-month follow-up*.

### Hemodynamic Response Function

**Figure 5:**
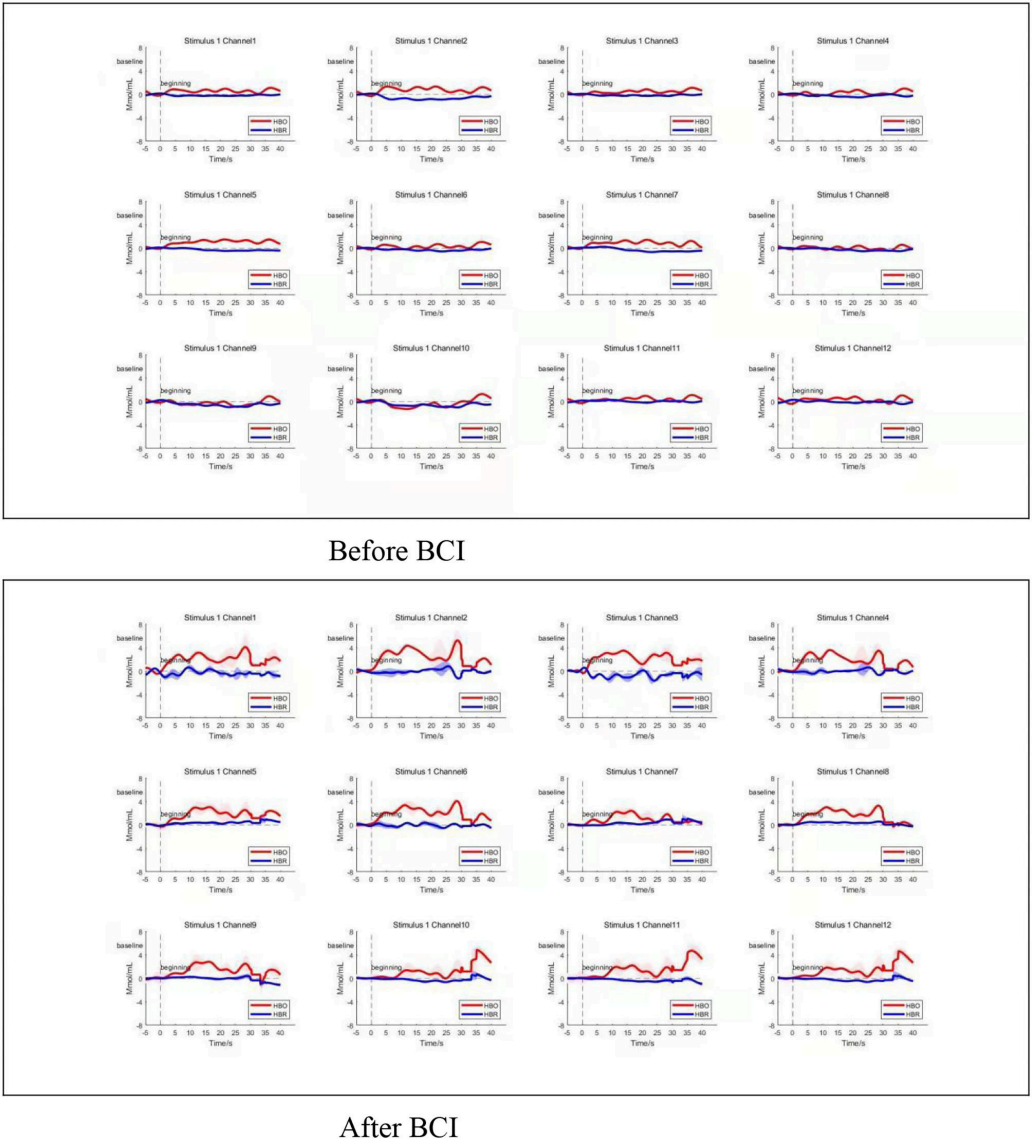
Average time series of HRF. Changes in average oxy-Hb amplitude increased at any time point in the whole four ROIs after BCI training during the grasping task (bottom panel). Channels 1–4 located in left M_1_, and channels 5–8 located in right M_1_. Channels 9 and 10 located in the left frontal polar cortex, and channels 11 and 12 located in the right frontal polar cortex. Top three panels show the HRF change before BCI training, bottom three panels show the HRF change after BCI training. HBO refers to oxy-Hb. HBR refers to deoxy-Hb.

Compared to the baseline, the averaged amplitude of HbO_2_ concentration increased after BCI training in the four ROIs significantly during the grasping task (Figure 5). Compared to the baseline, there was a significant increase in average Oxy-Hb amplitude at any time point in the whole four ROIs after BCI training during the grasping task.

### Resting-State FC Analysis

**Figure 6:**
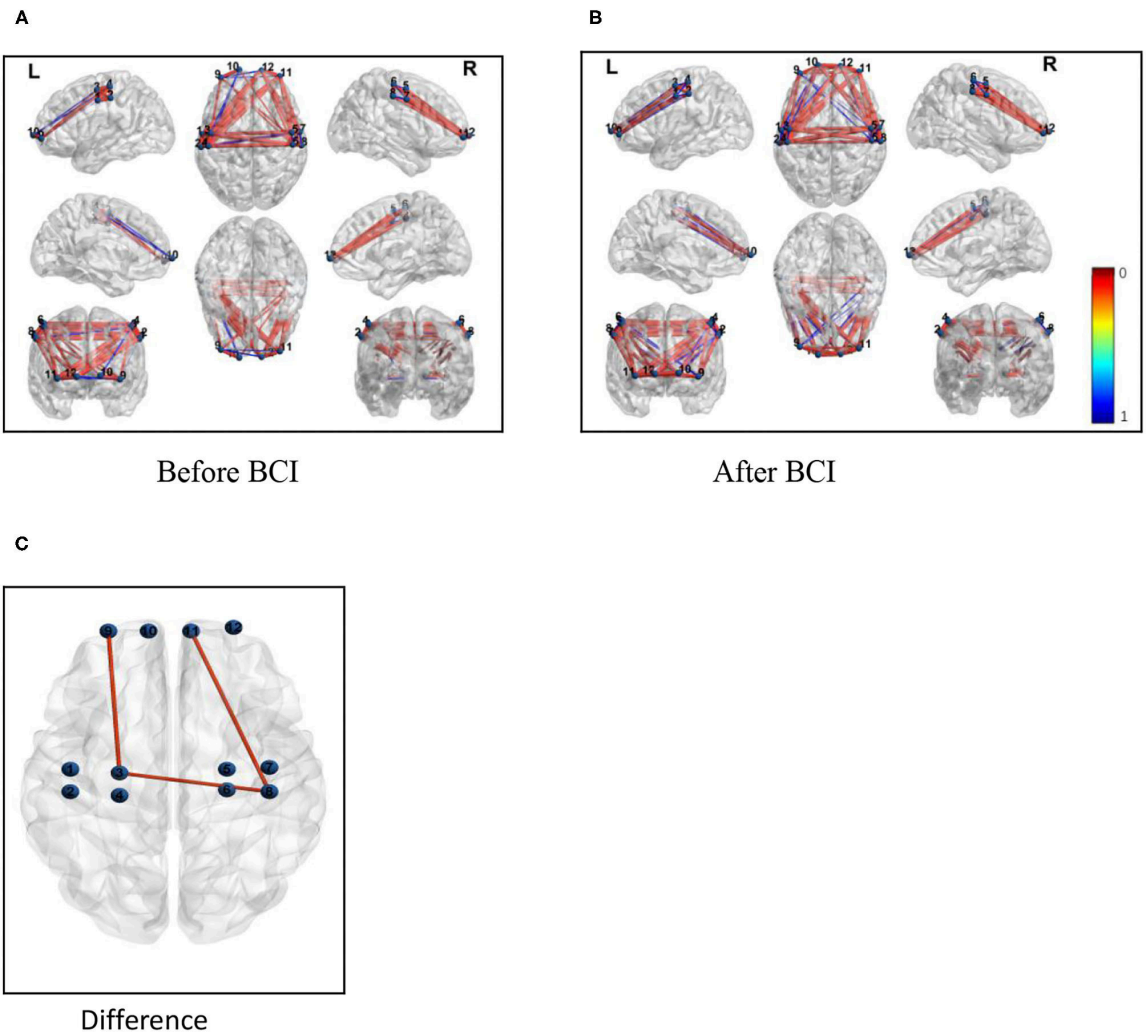
Functional connectivity (correlation) change measured after BCI training. Channels with differences in FC at baseline and the 1-month follow-up (Pearson correlation). **(A)** Correlations between each channel before BCI training. **(B)** Correlations between each channel after BCI training. **(C)** Correlations between the ipsilateral M_1_ and the ipsilateral frontal lobe and the contralateral M_1_ increased and the FC between the contralateral M_1_ and the contralateral frontal lobe was also increased.

For resting-state FC, the increased correlation between the ipsilateral motor cortex and ipsilateral frontal lobe was observed after BCI training (*Z* = 0.5835, *P* = 0.024; Figure 6B). Increased coherence between the ipsilateral motor cortex and contralateral motor cortex was also observed after BCI training (*Z* = 0.7964, *P* = 0.035; Figure 6C). Additionally, increased FC was measured between the contralateral motor cortex and contralateral frontal lobe after BCI training (*Z* = 0.7934, *P* = 0.028; Figure 6C). There was no significant difference in FC between the ipsilateral motor cortex and contralateral frontal lobe measured after BCI training (*Z* = 0.64, *P* = 0.067; Figure 6C).

## Discussion

This study evaluated the effects of a closed-loop neurofeedback BCI-robot system on the improvement of the motor functional recovery of the upper extremities, which was measured by the WMFT and FMA-UE test, as well as the FC between the motor and sensory systems in the brain. The findings on the neuroplastic effects of BCI training showed FC changes involving the contralateral hemisphere in task-related brain activation induced by BCI training rehabilitative therapy. Specifically, the examination of the fNIRS showed changes in the patterns of brain activation associated with the paralyzed hand grasping were observed with the administration of BCI therapy. The grasp of the impaired hand was accompanied by a more increasing connectivity pattern after BCI therapy between the ipsilesional primary motor cortex (M_1_) and the ipsilesional frontal cortex (Figure 6).

In this study, both the WMFT and the FMA scores were found to be significant using one-way ANOVA on this data after the BCI therapy. In controlled trials, when compared with control interventions, the results showed that BCIs had a significant effect on the improvement of upper extremity function (Ono et al., 2014). When comparing to standard robotic rehabilitation, it showed in the experimental group the average motor improvement was slightly more than in controls (Ang et al., 2014; Ono et al., 2014; Remsik et al., 2016). In single-group studies, all of them indicated significant improvements in the scores of the FMA-UE and other functional scales (Halder et al., 2011), and we obtained the similar research results. We noted that in this preliminary study, we did not design a control group, which used a pre- and post-intervention comparison to observe differences in clinical outcomes, which, first, could be sources of significant biasTo reduce bias, we optimized the BCI system used in this study, which removed the instruction automatically of the robotic hand and quantified the control recognition ability of the BCI system when the patient performed motion observation and motion imagery. In addition, we noted that the participants in the studies were at chronic stages, in which spontaneous recovery was less likely to occur (Soekadar et al., 2015; Remsik et al., 2016). However, some scholars suggested that different lengths of the BCI intervention might be another confounding factor (Ang et al., 2015b). In our study, all subjects completed these behavioral assessments, including post-therapy and 1-month FU assessments after 20 sessions of interventions. It may also be taken into consideration that both WMFT and FMA-UE are objective measurements of clinical function.

According to BCI system studies, patients in the chronic stage have the accuracy to perform MI with diverse feedback, including visual, kinesthetic, and/or proprioceptive feedback, even in those severe cases in whom volitional isolated finger movement is not possible. Serious movement dysfunction in the upper extremity requires more help, such as a robotic device to achieve hand functional movement (Pichiorri et al., 2015). Recently, more evidence has demonstrated that the motor function of patients with severe stroke could be promoted by BCI, in particular, distal hand function (Frolov et al., 2017). Compared with the sham group, the clinical performance above MCID in the BCI group could be observed. Another study (Ramos-Murguialday et al., 2013) included severely impaired subjects and designed a homogeneous sample with a good match by age, gender, paretic side, and motor impairment scores in the controlled trial. Subjects in both groups had a similar amount of BCI training, and the experimental group had higher muscle activity and showed a significant improvement in hand function. Furthermore, research work made by Shindo demonstrated that finger function and surface EMG activity have been improved without motor function (Shindo et al., 2011). All these findings suggest that BCI can be a promising strategy in the rehabilitation of chronic stroke.

Motor recovery after stroke relies on neural plasticity at both structural and functional levels. Rehabilitation training after stroke may strengthen neural connections in existing ones and/or lead to the formation of new neural pathways (Rathee et al., 2017). Some randomized controlled trials mentioned the mechanisms of neural responses to BCI training. One previous EEG study showed that desynchronization over the ipsilesional central area during MI tasks after BCI training has a greater response than pre-intervention, indicating higher activation of the motor area in the ipsilesional brain through BCI training (Mihara et al., 2013; Li et al., 2014). The changes in FC patterns during the grasp of the impaired hand associated with the intervention of BCI therapy suggest that there may be a neuroplastic response to the treatment between the motor system and the sensory system. Several previous studies showed neuroplasticity changes due to BCI intervention, including increasing activity in pre-motor cortex (PMC) or enhancement of ipsilesional connectivity in hemispheric EEG activity (Cervera et al., 2018). Particularly, an fNIRS study of patients with subcortical stroke also showed the activation of the PMC, which was considered to be associated with motor planning and the execution of goal-oriented actions (Sugawara et al., 2013). In addition, research work proved that BCI might activate some special brain cortex, including the prefrontal cortex, PMC, and posterior parietal cortex in some pre-post single-group designs (Halder et al., 2011). By using structural equation modeling of resting-state fMRI data, an investigation was made to explore the effective connectivity between motor control and motor execution. Connectivity from frontoparietal guidance systems to the motor network is diminished in stroke survivors (Inman et al., 2012). In our study, we found FC between ipsilesional M1 and frontal cortex might be enhanced after BCI training. This might improve the overall BCI performance. This result indicates an enhancement of synchronization during the neurological activity among the different cerebral regions after stroke (Figure 6). The increased FC in the brain regions supported that BCI training could improve the degree of the disturbance of the neurovascular activities of the brain. The brain activity changes examined by fNIRS may be a response to an objective measurement that is proven by standardized motor function tests.

Recently, a study has described the relevance between neuroplasticity and functional improvement in the MI-based BCI training system (Varkuti et al., 2013). The authors concluded that BCI training might strengthen neuroplasticity and lead to better recovery, due to MI-based BCI might control voluntary movement of the hand in the same neural mechanisms (Orihuela-Espina et al., 2013; Remsik et al., 2016). Additionally, the persistence of these changes up to 1-month FU after the intervention of BCI therapy indicated the possibility for lasting effects under the conditions of this rehabilitative approach. The brain has a certain degree of plasticity, which could be strengthened by using feedback information such as punishment or reward. By decoding and outputting the patient's neural information, BCI could control peripheral muscles and provide feedback to form a new “closed-loop pathway” between the central nervous system and the peripheral nervous system, and then promote the remodeling and recovery of brain function after stroke. Relying on the direct central intervention of the brain, it can further activate functional neuroplasticity and promote cortical remodeling (Birbaumer, 2006). Several studies have shown that (Hummel and Cohen, 2006) 63% of patients with hemiplegia have functional asymmetry after damage to the brain, which has widely been considered to have an unbeneficial effect on the patient's functional recovery. The more the asymmetry is, the worse the recovery of movement will be. A meta-analysis shows (Tang et al., 2015) that the asymmetric performance of the brain after stroke could be improved to a certain extent by BCI intervention. In the next study, we will focus on the inter-hemispheric and intra-hemispheric connections. BCI combined with external robots (e.g., exoskeletons and orthosis) or FES formed the closed-loop intervention mode and promoted better hand function recovery through inducing the activation of specific brain regions.

Nevertheless, the specific mechanisms of the BCI system underlying functional improvements remain largely unknown. In our study, BCI might promote the activation of ipsi or peri-lesional cortex, which effectively results in functional motor activity in the lesioned hemisphere, as was observed in the previous study through the BCI-FES system by scalp EEG analysis (Biasiucci et al., 2018). A meta-analysis of the effect of the BCI system in stroke recovery suggested movement intention and movement-dependent approaches of ipsilesional reorganization caused by BCI coinciding with better recovery in the chronic phases of stroke (Bai et al., 2020). Additionally, the heterogeneity of current BCI systems complicates attempts to elucidate their mechanisms, because it is likely that functional improvements rely on different strategies that target different aspects of neural circuitry. Besides, BCI improvement may also be influenced by effects from “non-motor” mechanisms (Simon et al., 2021), such as sustained exertion of effort, senses of achievement from controls of the BCI, or improvements in the mood by engaging with a challenging task.

This study has shown the effect on motor performance after BCI-hand robotic training. Additionally, we conducted an examination of neural activities by fNIRS. Changes in neural FC patterns were observed with BCI therapy in both motor and sensory areas in the ipsilesional cerebral in this population of patients with stroke. But there were some limitations and challenges to BCI research work. First, we must be careful of the small sample (*n* = 18) and heterogeneity of stroke survivors, including the different levels of motor impairment (moderate to severe) and lesions (cortical to subcortical). BCI might induce the consistent brain changes of pre-post intervention in these patients over the time course. We noted that although these research findings were promising, the scope of the conclusions was limited by the lack of a control group. Further randomized controlled trials with larger samples should also be designed strictly and performed in the next step to further verify this hypothesis. Second, though most studies about the BCI system tended to be efficient in the movement recovery for patients in the chronic stage after stroke, it is impossible that patients had the whole motivation to continue investing effort into trying to control the BCI without the therapist's assistance, which made it difficult to utilize this rehabilitative technology at home. Third, we assumed the non-motor mechanisms that contributed to the functional recovery during the control of the BCI system. In the further study, we attempt to improve scientific rigor and reproducibility in neurofeedback research.

In summary, BCI training is safe for patients after chronic stroke. More standardized studies should be done to better demonstrate and elucidate the effects of the BCI system therapy and to identify which protocols are the best therapy for different types of stroke. In particular, patients with severe motor deficits show greater recruitment of motor and non-motor areas such as the frontal areas of both the affected and unaffected hemispheres to strive for a full recovery.

## Data Availability Statement

The raw data supporting the conclusions of this article will be made available by the authors, without undue reservation.

## Ethics Statement

The studies involving human participants were reviewed and approved by the Human Ethics Committee of Shanghai Yangzhi Rehabilitation Hospital (#SBKT-2021-044). The patients/participants provided their written informed consent to participate in this study. Written informed consent was obtained from the individuals for the publication of any potentially identifiable images or data included in this article.

## Author Contributions

LL, LJ, and ZN designed the experiment. MJ and LZ conducted the measurements. LL, QZ, and DH participated in the data acquisition. LL supervised the whole process, data acquisition, analysis, manuscript revision, provided the scientific input, and contributed to the manuscript writing. LJ and ZN participated in the manuscript revision, supervised the whole process, and provided clinical input. All authors contributed to the article and approved the submitted version.

## Funding

This study was supported by the Shanghai Municipal Science and Technology Major Project (2021SHZDZX0100) and the Fundamental Research Funds for the Central Universities.

## Conflict of Interest

The authors declare that the research was conducted in the absence of any commercial or financial relationships that could be construed as a potential conflict of interest.

## Publisher's Note

All claims expressed in this article are solely those of the authors and do not necessarily represent those of their affiliated organizations, or those of the publisher, the editors and the reviewers. Any product that may be evaluated in this article, or claim that may be made by its manufacturer, is not guaranteed or endorsed by the publisher.
